# Altered Neuronal Activity Topography Markers in the Elderly with Increased Atherosclerosis

**DOI:** 10.3389/fnagi.2017.00216

**Published:** 2017-07-06

**Authors:** Takashi Shibata, Toshimitu Musha, Yukio Kosugi, Michiya Kubo, Yukio Horie, Naoya Kuwayama, Satoshi Kuroda, Karin Hayashi, Yohei Kobayashi, Mieko Tanaka, Haruyasu Matsuzaki, Kiyotaka Nemoto, Takashi Asada

**Affiliations:** ^1^Department of Neurosurgery, Stroke Center, Saiseikai Toyama HospitalToyama, Japan; ^2^Brain Functions Laboratory Inc.Yokohama, Japan; ^3^Department of Neurosurgery, Graduate School of Medicine and Pharmacological Science, University of ToyamaToyama, Japan; ^4^Department of Neuropsychiatry, Toho University Medical Center Sakura HospitalChiba, Japan; ^5^Department of Medical Course, Teikyo Heisei UniversityTokyo, Japan; ^6^Department of Neuropsychiatry, Institute of Clinical Medicine, University of TsukubaTsukuba, Japan; ^7^Department of Neuropsychiatry, University of Tokyo Medical and Dental UniversityTokyo, Japan

**Keywords:** EEG, vascular cognitive impairment, neuronal activity topography, atherosclerosis, elderly

## Abstract

**Background:** Previously, we reported on vascular cognitive impairment (VCI) templates, consisting of patients with VCI associated with carotid stenosis (>60%) using a quantitative electroencephalographic (EEG) technique called neuronal activity topography (NAT). Here using the VCI templates, we investigated the hypothesis that internal carotid artery–intima-media thickness (ICA–IMT) is associated with EEG spectrum intensity (sNAT) and spectrum steepness (vNAT).

**Methods:** A total of 221 community-dwelling elderly subjects were recruited. Four groups were classified according to quartiles of ICA–IMT as assessed by ultrasonography: control group A, normal (≤0.9 mm); group B, mild atherosclerosis (1−1.1 mm); group C, moderate atherosclerosis (1.2−1.8 mm); and group D, severe atherosclerosis (≥1.9 mm). EEG markers of power ratio index (PRI), and the binary likelihood of being in the VCI group vs. the that of being in control group A (*s*L_*x*:*VCI*−*A*_, *v*L_*x*:*VCI*−*A*_) were assessed, respectively. Differences in mean total scores for PRI, *s*L_*x*:*VCI*−*A*_, *v*L_*x*:*VCI*−*A*_, between control group A and the other groups were compared using Dunnett's test, respectively.

**Results:** The mean total scores of the PRI were 3.25, 3.00, 2.77, and 2.26 for groups A, B, C, and D, respectively. There was a significant decrease in the PRI in group D compared with group A (*P* = 0.0066). The mean total scores of the *s*L_*x*:*VCI*−*A*_ were −0.14, −0.11, −0.1, and −0.03 for groups A, B, C, and D, respectively. The *s*L_*x*:*VCI*−*A*_ in group D was significantly higher compared to that in group A (*P* < 0.0001). The mean total scores of the *v*L_*x*:*VCI*−*A*_ were −0.04,−0.01, 0.01, and 0.06 for group A, B, C, and D, respectively. The *v*L_*x*:*VCI*−*A*_ in group D and group C was significantly higher compared to that in group A, respectively (*P* < 0.0001, *P* = 0.02).

**Conclusion:** Community-dwelling elderly subjects in the increased carotid atherosclerosis of ICA–IMT (≥1.9 mm) were at greatest risk of an EEG change as assessed by NAT.

## Introduction

The carotid bifurcation and the proximal part of the internal carotid artery (ICA) are predilection sites for atherosclerotic plaques. Changes in the ICA–intima-media thickness (IMT) in this area, as assessed by ultrasonography, are a first sign of subclinical atherosclerosis (Polak et al., 2010; Bauer et al., 2012). Ultrasonography is an easily accessible and noninvasive method to measure different stages of the carotid artery atherosclerotic process, and it is widely used in clinical assessments and for epidemiological and clinical research (Arntzen and Mathiesen, 2011).

Carotid disease is a known risk factor for stroke and vascular cognitive impairment (VCI), but the relationship between carotid artery stenosis and cognitive function in asymptomatic people is unclear. The role of subclinical atherosclerosis in cognitive function can be studied by ultrasound measurement of the carotid arteries and neuropsychological tests. Previous studies indicate that patients with carotid stenosis have markedly poorer scores on cognitive tests compared with control subjects (Rao, 2001; Johnston et al., 2004; Mathiesen et al., 2004; Arntzen and Mathiesen, 2011). One cross-sectional study found that subjects with carotid stenosis (>35%) had lower levels of performance on cognitive function tests than subjects without stenosis (Mathiesen et al., 2004). In a cohort from the Cardiovascular Health Study (Haan et al., 1999), cognitive decline was markedly increased in subjects with an ICA–IMT > 2.01 mm. Most patients with subclinical carotid atherosclerosis have only minor impairments of cognitive function, and standard tests (e.g., the Mini-Mental State Examination: MMSE) are not sufficiently sensitive to detect such impairments. However, early detection of VCI is of particular importance because pharmacological intervention to prevent or delay dementia will prove effective for most patients with subclinical carotid atherosclerosis.

Electroencephalographic (EEG) signals are generated by electrical activity in the brain and are rich in information regarding cerebral function. In 2013, Musha et al developed a neuroimaging tool called Neuronal Activity Topography (NAT), which gives direct information on neuronal activity using quantitative EEG analysis (Musha et al., 2013). This tool categorizes cerebral neuronal activity by EEG spectrum intensity (sNAT) and spectrum steepness (vNAT). NAT consists of 210 submarkers referring to 10 frequency components ranging from 4 to 20 Hz (more precisely, from 4.7 to 18.8 Hz). Each submarker has its own role in the characterization of cerebral neuronal activity, which is represented by the 210-dimensional NAT spaces. The NAT system has been used to detect Alzheimer's disease (AD) and to discriminate AD from other forms of dementia, VCI, dementia with lewy bodies, and is currently undergoing testing for its practical use in the clinical setting (Musha et al., 2002, 2004, 2013). Recently, we reported on VCI templates, consisting of patients with VCI associated with moderate carotid stenosis (>60%) and normal controls (NLc) (Shibata et al., 2014). In brief, the binary likelihood of being in the VCI group vs. that of being in NLc group (*s*L_*x*:*VCI*−*NLc*_, *v*L_*x*:*VCI*−*NLc*_) was assessed in each of the sNAT and vNAT spaces. Separation of the VCI group and NLc group was made with a sensitivity of 92 and 88%, as well as a false-positive rate of 8 and 12% for *s*L_*x*:*VCIc*−*NLc*_ and *v*L_*x*:*VCI*−*NLc*_, respectively. Therefore, the VCI templates based on NAT might be applied to community-dwelling elderly people to detect an EEG change reflecting VCI.

If EEG markers of NAT combined with ICA–IMT measurement could detect cognitive decline in subclinical atherosclerosis, then a combination of EEG and ultrasonography could be used to detect cognitive decline during routine medical checkups, because both EEG and ultrasonography are inexpensive, reliable, and noninvasive. To the best of our knowledge, the relationship between EEG finding and ICA–IMT has not yet been explored in elderly subjects. In the present study, using the VCI templates previously obtained from Toyama city, we investigated the hypothesis that increased ICA–IMT are associated with altered EEG markers of NAT for community-dwelling elderly people in Tsukuba city.

## Subjects and methods

### Characteristics of survey subjects

The present investigation is part of the Tsukuba epidemiological investigation project for the prevalence of dementia among inhabitants older than 65 years of age in Tsukuba, Ibaraki, Japan. This study was approved by the ethical committees from the University of Tsukuba (Tsukuba, Japan). All subjects gave written informed consent in accordance with the Declaration of Helsinki. As part of this project, a three-phase survey was carried out in the Tsukuba area. The survey protocol has been described in detail in a previous report (Ikejima et al., 2012). Between February 2012 and October 2012, 221 community-dwelling elderly subjects were enrolled. The 221 subjects underwent ultrasonography, EEG recording, and magnetic resonance imaging (MRI; 1.5T ECHELON RX; Hitachi Medical Corporation, Tokyo, Japan). Deep and periventricular white matter hyperintensities (WMH) were coded from 0 to 3 according to the Fazekas scale (Fazekas et al., 1987). Each EEG finding was independently interpreted by two EEG specialists who were blind to other data about the patients except their age and sex. All subjects underwent the MMSE to screen for cognitive function (cut-off score for cognitive impairment = 26/27).

### Characteristics of VCI group (Shibata et al., 2014)

The selected 55 VCI inpatients previously admitted at our hospital in Toyama city included 47 men and 8 women aged 58–87 years [mean ± standard deviation (SD), 72.6 ± 7.1 years]. Patients were selected based on the following criteria: (i) evidence of unilateral carotid stenosis of >60% (symptomatic or asymptomatic) confirmed with conventional angiography or computed tomography (CT) angiography and a degree of carotid artery stenosis determined using the criteria of the North American Symptomatic Carotid Endarterectomy Trial and (ii) mild cognitive impairment, considered as a score of <90 (low average) on the Repeatable Battery for the Assessment of Neuropsychological Status (RBANS) (Takaiwa et al., 2006, 2009). Of the 55 VCI patients with proximal carotid stenosis, 23 had right-sided lesions (stenosis or occlusion), 19 had left-sided lesions, and 13 had bilateral lesions. The mean score (± SD) on the MMSE and RBANS for the 55 VCI patients was 27.1 ± 1.5 and 74.5 ± 13.6, respectively. Exclusion criteria included (i) evidence of a previous major stroke and brain damage revealed through MRI; (ii) rapidly evolving symptoms with any hemiparesis, aphasia, and apraxia; and (iii) evidence of dementia, considered as MMSE score of <24, (iv) history of cerebral surgery, obvious psychiatric or neurological disorders, or (v) uncontrolled or malignant general complications. All VCI patients had good levels of daily living activities.

**Figure 1:**
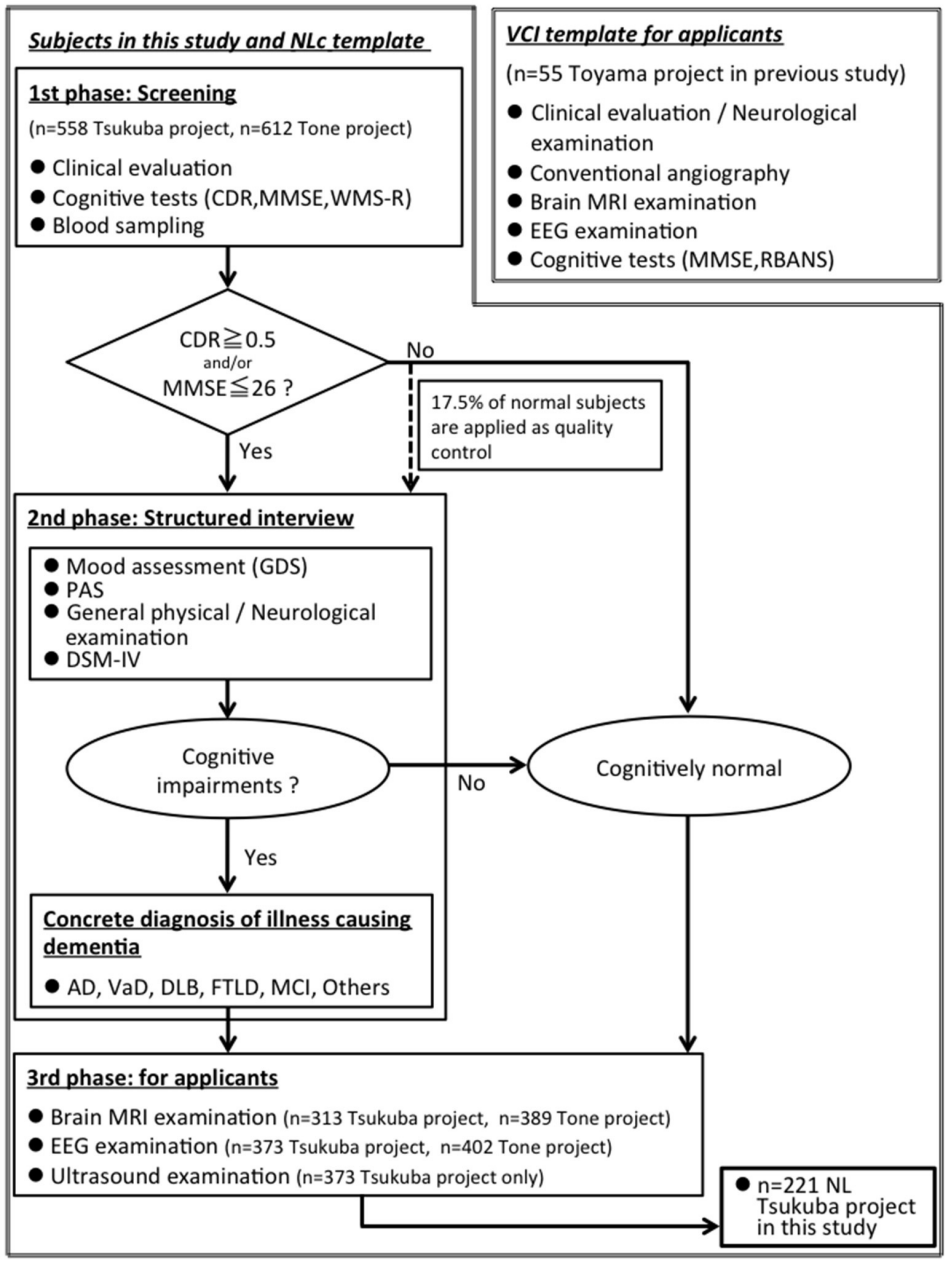
A flow chart for understanding two groups: (1) elderly group: community-dwelling elderly subjects from Tsukuba city, *n* = 221, (2) VCI group: VCI patients from Toyama city (Shibata et al., 2014), *n* = 55. MMSE, Mini-Mental State Examination; CDR, Clinical Dementia Rating; WMS-R, “logical memory A” from the Wechsler Memory Scale-Revised; PAS, Psychogeriatric Assessment Scales; RBANS, Repeatable Battery for the Assessment of Neuropsychological Status; GDS, Geriatric Depression Scale-Short Form; DSM-IV, Diagnostic and Statistical Manual of Mental Disorders, 4th edition; AD, Alzheimer disease; VaD, Vascular dementia; DLB, Dementia with Lewy Bodies; FTLD, FrontoTemporal Lober Degeneration; MCI, Mild Cognitive Impairment.

We showed a flow chart for understanding two groups: (1) elderly group: community-dwelling elderly subjects from Tsukuba city, *n* = 221, (2) VCI group: VCI patients from Toyama city, *n* = 55 (Figure 1).

### EEG recording and preprocessing

Electroencephalographic (EEG) recordings (EEG-1200/9100; Nihon Kohden Corporation, Tokyo, Japan) were performed in an awake, resting state with eyes closed for 10 min. Scalp potentials were recorded with 21 electrodes arranged according to the international 10–20 System (Fp1, Fp2, F3, F4, F7, F8, Fpz, Fz, T3, T4, T5, T6, C3, C4, Cz, P3, P4, Pz, O1, O2, and Oz). The contact impedance between the electrode and the scalp was < 50 kΩ. The reference electrode was on the right earlobe, and then the average reference for NAT analysis is computed as a mean of all electrodes. The raw EEG data were recorded at 0.08~300 Hz with 1,000 Hz sampling rate, and then the converted EEG data for NAT analysis were sampled at 200 Hz per channel and bandpass filtered to pick up signals in a frequency range of 4–20 Hz, which minimizes the effect of artifacts contaminating the recorded signals. The recorded signal sequence was divided into 0.64-s segments. To make the EEG data as high quality as possible and exclude the artifacts, each EEG finding was independently interpreted by EEG specialist who was blind to other data about the subjects except their age and sex. We carefully avoided particular epochs containing ocular movements, baseline shifts, drowsiness signs, and muscle or cardiac contamination.

### EEG data analysis

The average power ratio index (PRI) was calculated for the 221 subjects by dividing the total sum of low frequency (4–8 Hz) power spectrum by the total sum of high frequency (8–20 Hz) power spectrum at each electrode (Cz, C3, C4, Pz, P3, P4, Oz, O1, and O2), respectively, because of an efficient calculation of dominant parieto-occipital rhythm prior to *Normalized Power Spectrum (NPS)* analyses using the following formula:

(1) $$\begin{array}{c} PRI\equiv \Sigma PS_{h}/\Sigma PS_{l} \end{array}$$

The *PS*_*l*_ and *PS*_*h*_ represent power spectrum of low frequency (4–8 Hz) and that of high frequency (8–20 Hz), respectively.

The mathematical background of NAT (Brain Functions Laboratory, Inc., Yokohama, Japan) was previously described in detail (Musha et al., 2013).

1) Normalized Power Spectrum

We are going to derive dimensionless markers from the EEG signals which characterize stochastic nature of the cerebral neuronal activities generating these signals. The discrete power spectrum PS consists of the ten frequency components ${\langle {\left|X_{j,m}\right|}^{2}\rangle }_{seg}$ for *m* = 3–12 on signal channel *j*. The subscript attached to the averaging symbol denotes, hereafter, that the averaging is performed on this variable. In the present case, the averaging is performed across all of the segments. Dependence of the EEG signal level on individual subject is eliminated by normalizing it to its mean level. The NPS consists of 10 (*m* = 3–12) such components NPS_*j, m*_ defined as,

(2) $$NPS_{j,m}={\langle |X_{j,m}{|}^{2}\rangle }_{seg}/{\langle |X_{j,m^{\prime }}{|}^{2}\rangle }_{seg,m^{\prime }}\;\cdot$$

They make a set of 10 submarkers for each signal channel. This marker characterizes the fractional partition of the EEG power over the 10 frequency components.

Another role of the collective neuronal activities is the signal transmission through the neuronal networks. The biological signal is encoded as the modulation of the occurrence rate of neuronal activities. The organized modulation mode introduces coherence in the collective neuronal activities, and the coherence causes spiky variations of the power spectrum. The modulation is characterized by a ratio *p*_*j, m*_ between the adjacent power components given as $p_{j,m}={\langle {\left|x_{j,m+1}\right|}^{2}\rangle }_{seg}/{\langle {\left|x_{j,m}\right|}^{2}\rangle }_{seg}$. Such ratios derive the 10 (*m* = 3–13) dimensionless submarkers NPV_*j, m*_ on the signal channel j,

(3) $$NPV_{j,m}=\frac{4p_{j,m}}{{\left(1+p_{j,m}\right)}^{2}}\;\cdot$$

2) Zero-Level Resetting

Averages (NPS_*j l,m*_)_*seg, jl*_ and (NPV_*j l,m*_)_*seg, jl*_ across the signal channels are left with offset values. The offset values lower the quality of discrimination between different brain diseases, and new markers sNAT_*j, m*_ and vNAT_*j, m*_ are introduced after removing the offset as,

(4) $$\begin{array}{c} sNAT_{j,m}\equiv {\langle NPS_{j,m}\rangle }_{seg}- \end{array}{\langle NPS_{j\prime ,m}\rangle }_{seg,j\prime }$$

(5) $$vNAT_{j,m}\equiv {\langle NPV_{j,m}\rangle }_{seg}-{\langle NPV_{j^{\prime },m}\rangle }_{seg,j^{\prime }}\cdot$$

3) Likelihood

Briefly, a template marker sNAT map is prepared from data for 55 previously diagnosed VCI patients using the mean and SD of a number of sNAT states. The sNAT state of a test subject was assigned to a point in the 210-dimensional sNAT space, whereas the template state of the VCI patients was assigned to another point in this space. The states of patients with VCI are distributed within these spaces making clusters around their mean states, which are regarded as the template states $sNAT_{j,m}^{VCI}$ together with the standard deviations $s\sigma_{j,m}^{VCI}$ around them. $sNAT_{j,m}^{VCI}$ of VCI is defined as $sZ_{j,m}^{x:VCI}$ which is

(6) $$sZ_{j,m}^{x:VCI}\equiv \left(sNAT_{j,m}^{x}-sNAT_{j,m}^{VCI}\right)/s\sigma_{j,m}^{VCI}$$

The likelihood of the test subject *x* being in the VCI group, *sL*_*x*:*VCI*_, was given as a function of the effective distance between the two points properly normalized in terms of the mean and SD related to the VCI template. The likelihood *sL*_*x*:*VCI*_ of a test subject x to be in VCI is defined as,

(7) $$sL_{x:VCI}\equiv \exp{\langle -{\left(sZ_{j,m}^{x:VCI}\right)}^{2}\rangle }_{j,m}$$

The differential (binary) likelihood of being in the VCI group vs. the control group A, *sL*_*x*:*VCI*−*A*_ was defined as,

(8) $$\begin{array}{r} sL_{x:VCI-A}\equiv sL_{x:\text{VCI}}-sL_{x:A} \end{array}$$

Similarly, another differential likelihood in reference to vNAT was introduced as *vL*_*x*:*VCI*−*A*_. The subject was more likely to be in the VCI group than in the control group A when this value was more positive and vice versa.

### Ultrasonography protocol

The same sonographer, who was blinded to the subjects' case status and risk factor levels, carried out all ultrasound examinations. High-resolution B-mode ultrasonography of the ICA was performed with an EUB-5500 ultrasound machine (Hitachi Medical Corporation, Tokyo, Japan). Subjects were examined in the supine position for about 15–20 min. Longitudinal images of the ICA were obtained by combined B-mode and color Doppler ultrasound examinations. With this technique, two parallel echogenic lines separated by an anechoic space can be visualized at the level of the artery wall. These lines are generated by the blood-intima and media-adventitia interfaces. The distance between the two lines gives a reliable index of the thickness of the intima-media complex. The posterior (far) wall IMT was measured with the electronic calipers of the ultrasound machines, as described by Pignoli et al. (1986). On a longitudinal, 2-dimensional ultrasound image of the ICA, images of the posterior wall are displayed as two bright white lines separated by a hypoechoic space. The distance between the leading edge of the first bright line and the leading edge of the second bright line indicates the IMT. We assessed the maximum IMT, which was defined as the single thickest part of the wall among the near and far right and left walls of the ICA.

### Statistical analysis

All data were analyzed using JMP® 12 (SAS Institute Inc., Japan). Mean ± SD values of age, total MMSE score, and years of education were used as descriptive measures of normally distributed variables. The results were compared among the 4 groups included VCI group by analysis of Dunnett's test as control group A. Tukey's HSD (honest significant difference) was used for quantitative variables (age, total MMSE score and years of education), and the χ^2^ test was used for categorical variables (Fazekas score, EEG finding). Linear regression analysis among PRI, the binary likelihoods *s*L_*x*:*VCI*−*A*_ and *v*L_*x*:*VCI*−*A*_, and the ICA−IMT was performed. Differences with a *P*-value of < 0.05 were considered statistically significant.

## Results

The clinical characteristics of the 221 study subjects grouped according to quartiles of ICA−IMT are shown in Table 1. The 221 subjects included 110 men and 111 women with a mean (± SD) age of 74.9 ± 6.5 years. The mean MMSE score (± SD) was 28.8 ± 1.2, and the mean education period (± SD) was 12.7 ± 3.1 years. The quartiles of ICA−IMT were defined based on the maximum ICA−IMT and were 1, 1.2, and 1.9 mm. The groups were as follows: Group A, normal (ICA−IMT ≤0.9 mm); group B, mild atherosclerosis (1−1.1 mm); Group C, moderate atherosclerosis (1.2−1.8 mm); and Group D, severe atherosclerosis (≥1.9 mm). There were no significant differences in age, total MMSE score, years of education among the four groups. Although the cerebral white matter lesions on Fazekas class four and borderline on EEG findings were associated with an increased IMT, there were no significant differences in Fazekas score and EEG finding among the four groups (χ^2^ test).

**Table 1:** Clinical and radiological characteristics of the study population.

	**Group A**	**Group B**	**Group C**	**Group D**
Subjects	57	49	54	61
Sex (men/women)	18/39	27/22	30/24	35/26
Age	73.6 ± 6.2	74.4 ± 6.1	74.8 ± 6.2	76.6 ± 6.9
MMSE	28.9 ± 1.3	28.6 ± 1.1	28.9 ± 1.3	28.7 ± 1.2
Education	12.6 ± 2.8	13.1 ± 3.3	12.2 ± 3.2	12.8 ± 2.9
Max-IMT(Mean ± SD) mm	0.83 ± 0.13	1.07 ± 0.14	1.49 ± 0.31	2.79 ± 0.83
Max-IMT(Minimum^—^Max)	0.5–0.9	1–1.1	1.2–1.8	1.9–5.6
**EEG**				
Within Nomal Limits	47	34	41	42
No abnormality	2	7	2	2
Borderline	3	6	10	12
Sligtly slow abnormality	4	2	1	5
Moderately slow abnormality	1	0	0	0
**FAZEKAS CLASS**				
0	15	16	9	15
1	14	12	12	17
2	14	10	19	21
3	0	0	2	4

*Mean values ± standard deviation of demographic characteristics (age, MMSE score, years of education, and ICA–IMT, EEG finding, Fazekas score on MRI). Age and education are expressed in years. Group A, normal (ICA–IMT ≤ 0.9 mm); Group B, mild atherosclerosis (1–1.1 mm); Group C, moderate atherosclerosis (1.2–1.8 mm); Group D, severe atherosclerosis (≥1.9 mm). MMSE, Mini-Mental State Examination; IMT, intima-media thickness; and SD, standard deviation*.

**Figure 2:**
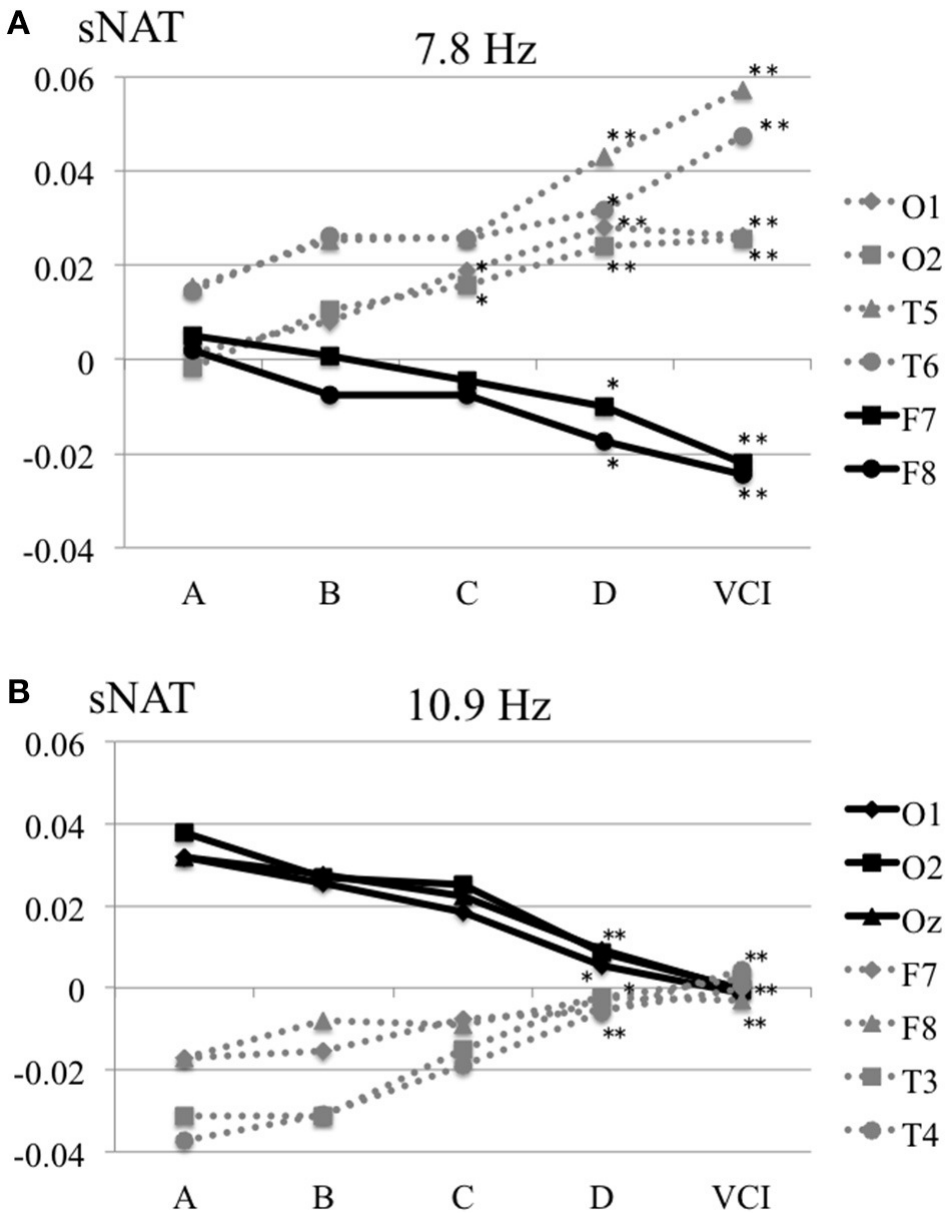
**(A)** Mean sNAT plot at 7.8 Hz frequency range over occipital-temporal areas (O1, O2, T5, T6) and frontal areas (F7, F8) for group A, group B, group C, group D, VCI group, respectively. **(B)** Mean sNAT plot at 10.9 Hz frequency range over occipital areas (O1, O2, Oz) and fronto-temporal areas (F7, F8, T3, T4) for group A, group B, group C, group D, VCI group, respectively. ^*^P <0.05, ^**^P < 0.01 (Dunnett 's test as control group A).

**Figure 3:**
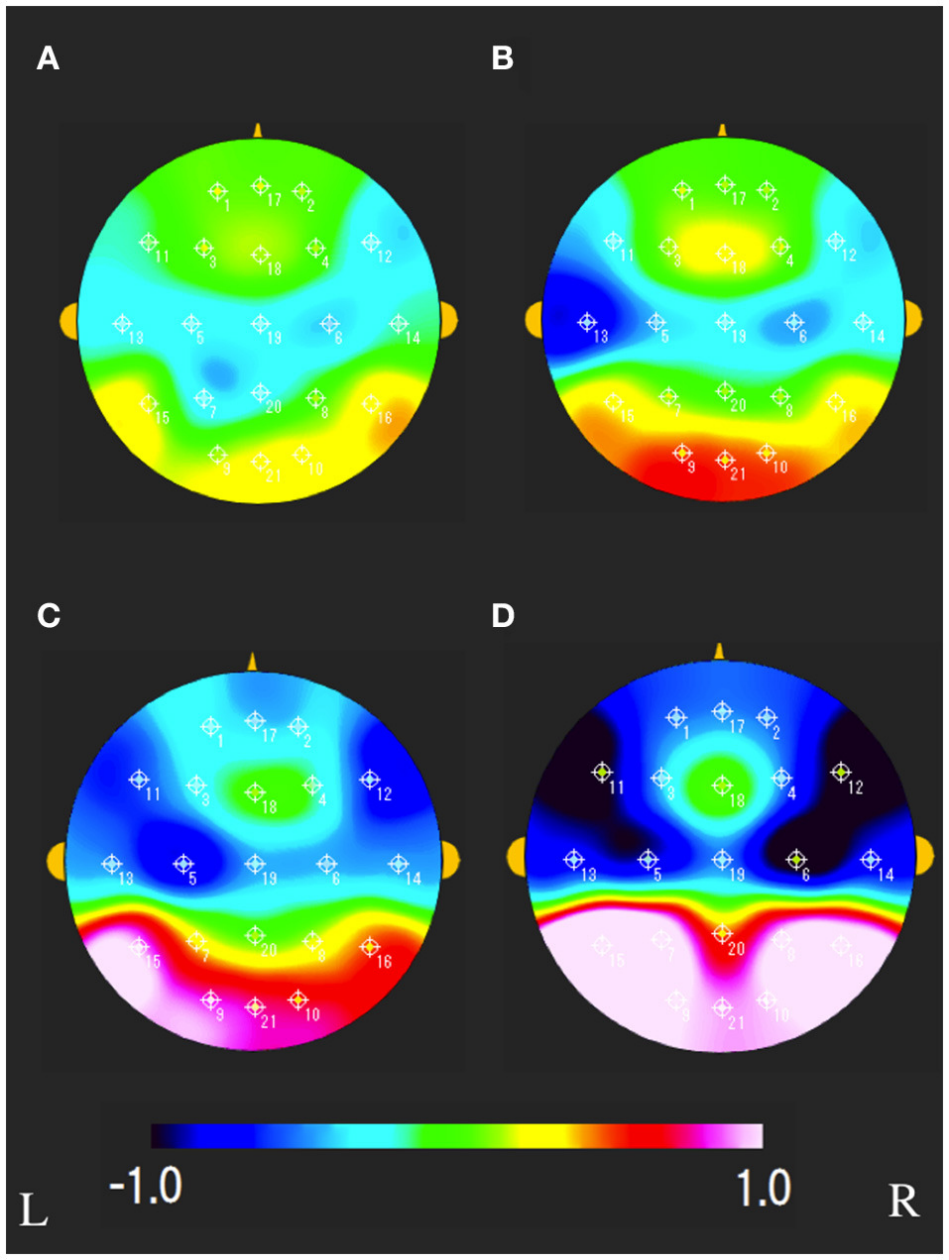
Mean sNAT map at 7.8 Hz frequency range for **(A)** group B, **(B)** group C, **(C)** group D, **(D)** VCI group, respectively. The colorbar indicates the range of z-scores in comparison with control group A: Green indicates normal spectrum intensity. Red and blue indicate hyperspectrum intensity and hypospectrum intensity, respectively. L, left; R, right.

The characteristics distributions of sNAT in the elderly with increased IMT have appeared at a specific frequency range of 7.8 Hz, 10.9 Hz as follows. (1) At theta frequency of 7.8 Hz, there were statistically significant increased activities over occipital-temporal areas (O1, O2, T5, T6, F7, and F8) and decreased activities over bilateral frontal areas (F7, F8) in comparison with control group A (Figure 2A). Mean z-score sNAT map at 7.8 Hz frequency range was shown for group B, group C, group D, VCI group in comparison with control group A, respectively (Figure 3). (2) At alpha frequency range of 10.9 Hz, there were statistically significant decreased activities over occipital areas (O1, O2, and Oz) and increased activities over bilateral fronto-temporal areas (F7, F8, T3, and T4) in comparison with control group A (Figure 2B).

**Figure 4:**
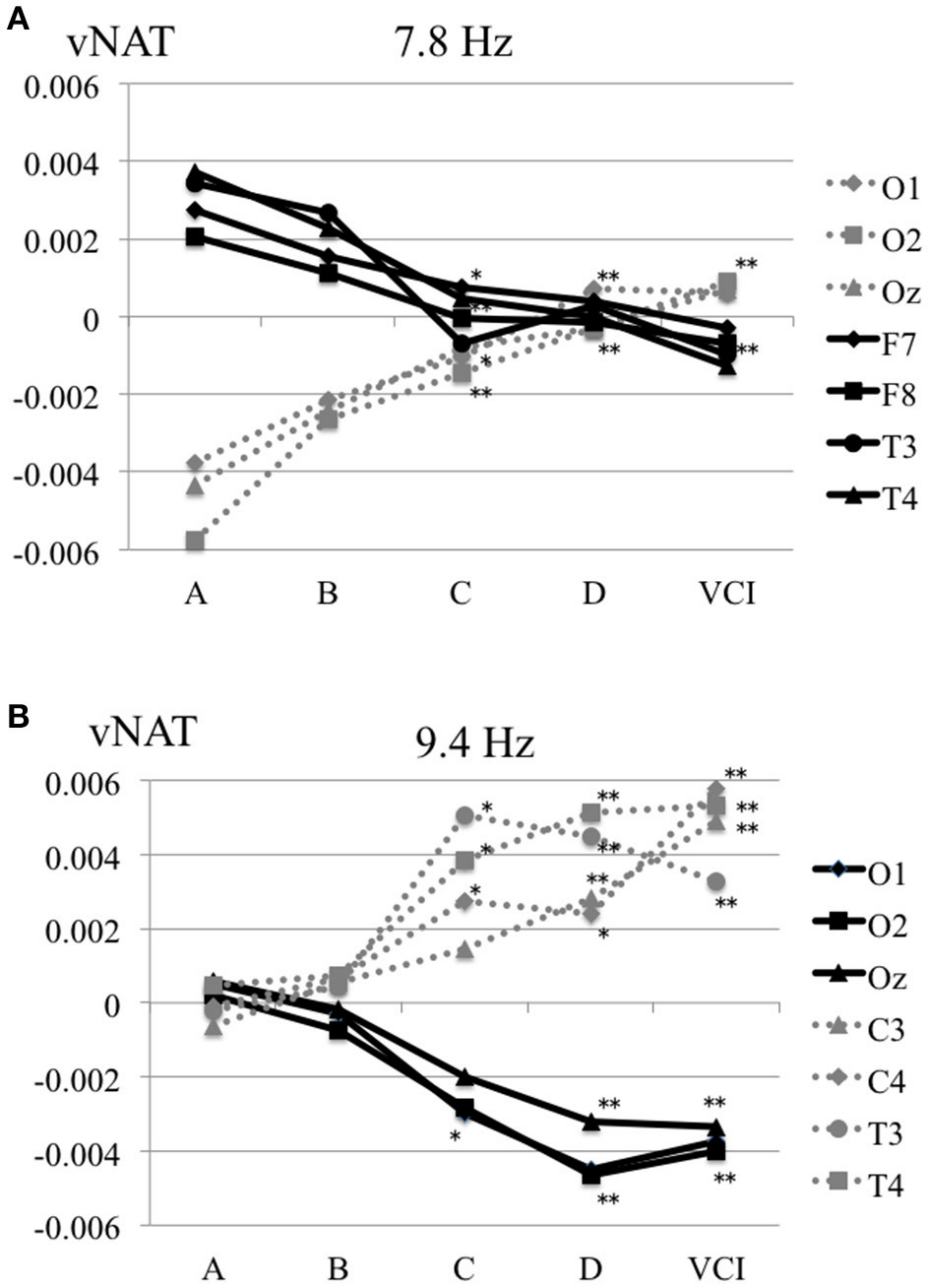
**(A)** Mean vNAT plot at 7.8 Hz frequency range over occipital areas (O1, O2, Oz) and fronto-temporal areas (F7, F8, T3, T4) for group A, group B, group C, group D, VCI group, respectively. **(B)** Mean vNAT plot at 9.3 Hz frequency range over occipital areas (O1, O2, Oz) and centro-temporal areas (C3, C4, T3, T4) for group A, group B, group C, group D, VCI group, respectively. ^*^P < 0.05, ^**^P < 0.01 (Dunnett 's test as control group A).

**Figure 5:**
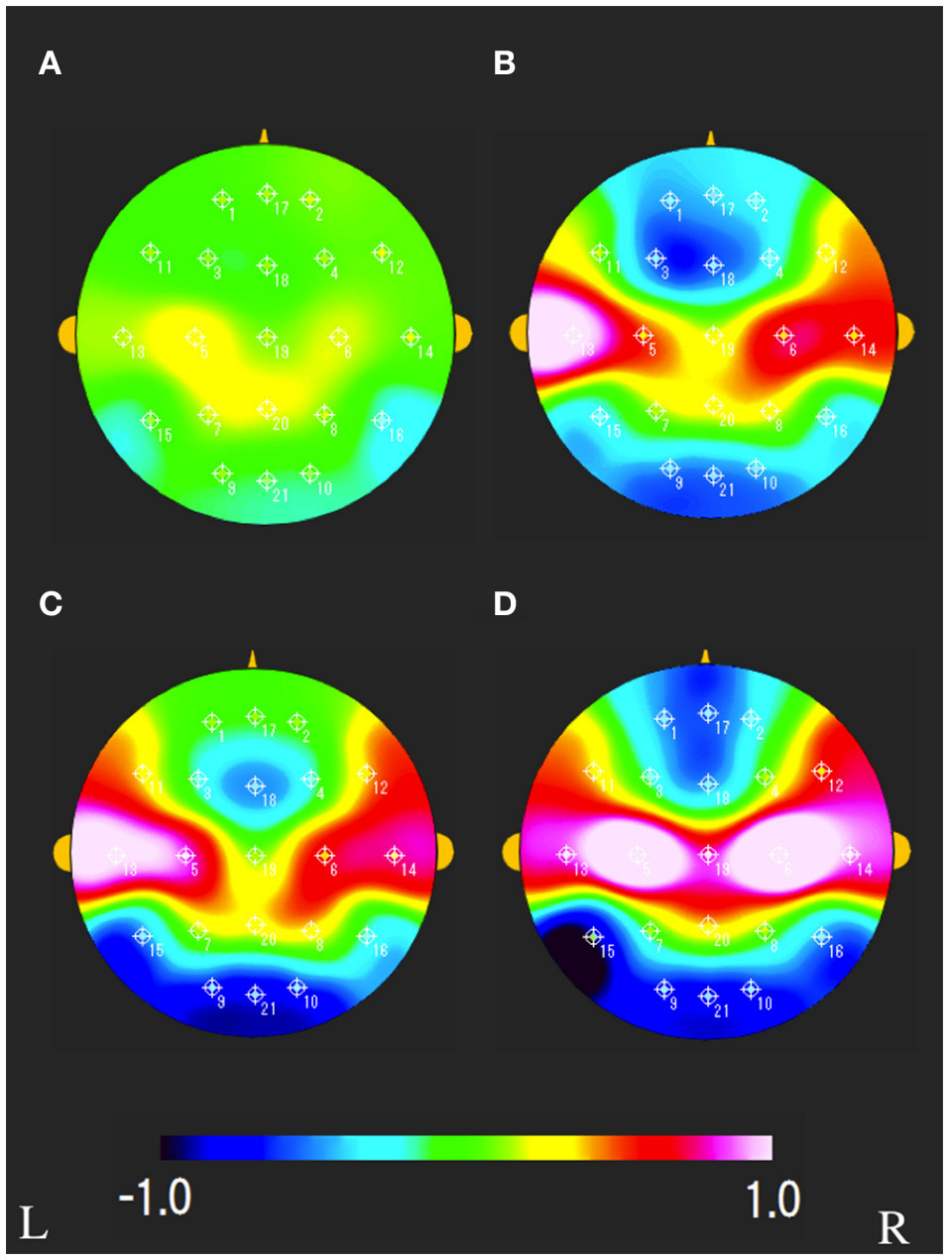
Mean vNAT map at 9.3 Hz frequency range for **(A)** group B, **(B)** group C, **(C)** group D, **(D)** VCI group, respectively. The colorbar indicates the range of z-scores in comparison with control group A: Green indicates normal spectrum steepness. Red and blue indicate spectrum blur and spectrum sharpness, respectively. L, left; R, right.

The characteristics distributions of vNAT in the elderly with increased IMT have appeared at a specific frequency range of 7.8 Hz, 9.4 Hz, respectively as follows. (1) At theta frequency range of 7.8 Hz, there were statistically significant increased activities over occipital areas and decreased activities over bilateral fronto-temporal areas in comparison with control group A (Figure 4A). (2) At alpha frequency range of 9.4 Hz, there were statistically significant decreased activities over occipital areas and increased activities over bilateral parieto-temporal areas in comparison with control group A (Figure 4B). Mean z-score vNAT map at 9.4 Hz frequency range was shown for group B, group C, group D, VCI group in comparison with control group A, respectively (Figure 5).

**Figure 6:**
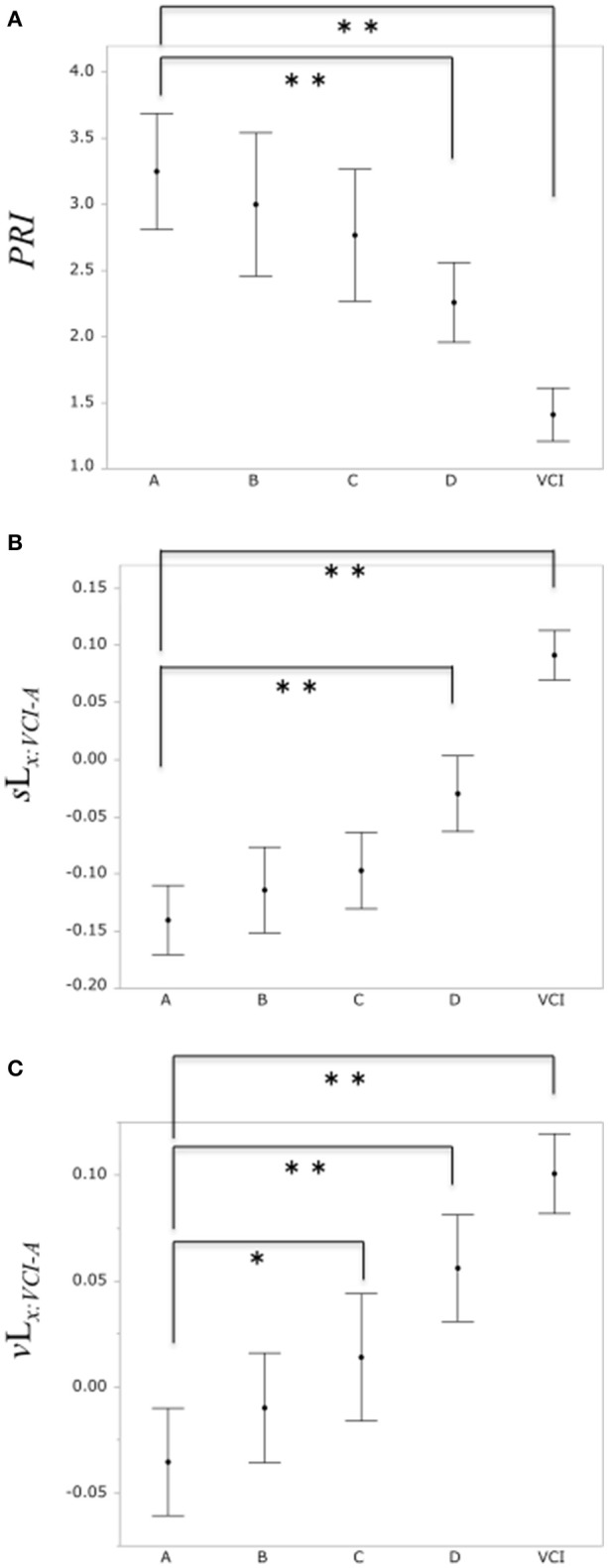
**(A)** Comparison of PRI among the control group A and subclinical atherosclerosis groups (B, C, D), VCI group, respectively. **(B)** Comparison of the binary likelihood *s*L_*x*:*VCI*−*A*_ among the control group (A) and subclinical atherosclerosis groups (B, C, D), VCI group, respectively. **(C)** Comparison of the binary likelihood *v*L_*x*:*VCI*−*A*_ among the normal group (A) and subclinical atherosclerosis groups (B, C, D), VCI group, respectively. Each filled circle indicates the mean of the PRI, *s*L_*x*:*VCI*−*A*_, *v*L_*x*:*VCI*−*A*_, respectively, and each vertical line indicates 95% confidence intervals. ^*^P < 0.05, ^**^P < 0.01.

A comparison of the PRI among the normal group A and the subclinical atherosclerosis groups (B, C, D), VCI group is shown in Figure 6A. The mean total scores (± SD) for the PRI were 3.25 ± 1.64, 3.00 ± 1.87, 2.77 ± 1.8, 2.26 ± 1.17, and 1.41 ± 0.69 for group A, B, C, D, and VCI, respectively. In results of Dunnett's test as control group A, there was a significant decrease of the PRI in the group D and VCI compared with the normal group A, respectively (*P* = 0.0018, *P* < 0.0001).

Comparison of the binary likelihood *s*L_*x*:*VCI*−*A*_ among the control group A and the subclinical atherosclerosis groups (B, C, D), VCI group is shown in Figure 6B. The mean total scores (± SD) for the binary likelihood *s*L_*x*:*VCI*−*A*_ were −0.14 ± 0.1, −0.11 ± 0.13, −0.1 ± 0.12, −0.03 ± 0.13, and 0.09 ± 0.08 for group A, B, C, D, and VCI, respectively. In results of Dunnett's test as control group A, there was a significant increase in the binary likelihood *s*L_*x*:*VCI*−*A*_ in the group D and VCI in comparison with control groups A, respectively (*P* < 0.0001, *P* < 0.0001).

Comparison of the binary likelihood *v*L_*x*:*VCI*−*A*_ among the control group A and the subclinical atherosclerosis groups (B, C, D), VCI group is shown in Figure 6C. The mean total scores (± SD) for the binary likelihood *v*L_*x*:*VCI*−*A*_ were −0.04 ± 0.09, −0.01 ± 0.09, 0.01 ± 0.1, 0.06 ± 0.1, and 0.1 ± 0.07 for groups A, B, C, D, and VCI, respectively. In results of Dunnett's test as control group A, there was a significant increase in the binary likelihood *v*L_*x*:*VCI*−*A*_ in group C, D, and VCI in comparison with control group A, respectively (*P* = 0.021, *P* < 0.0001, *P* < 0.0001).

**Figure 7:**
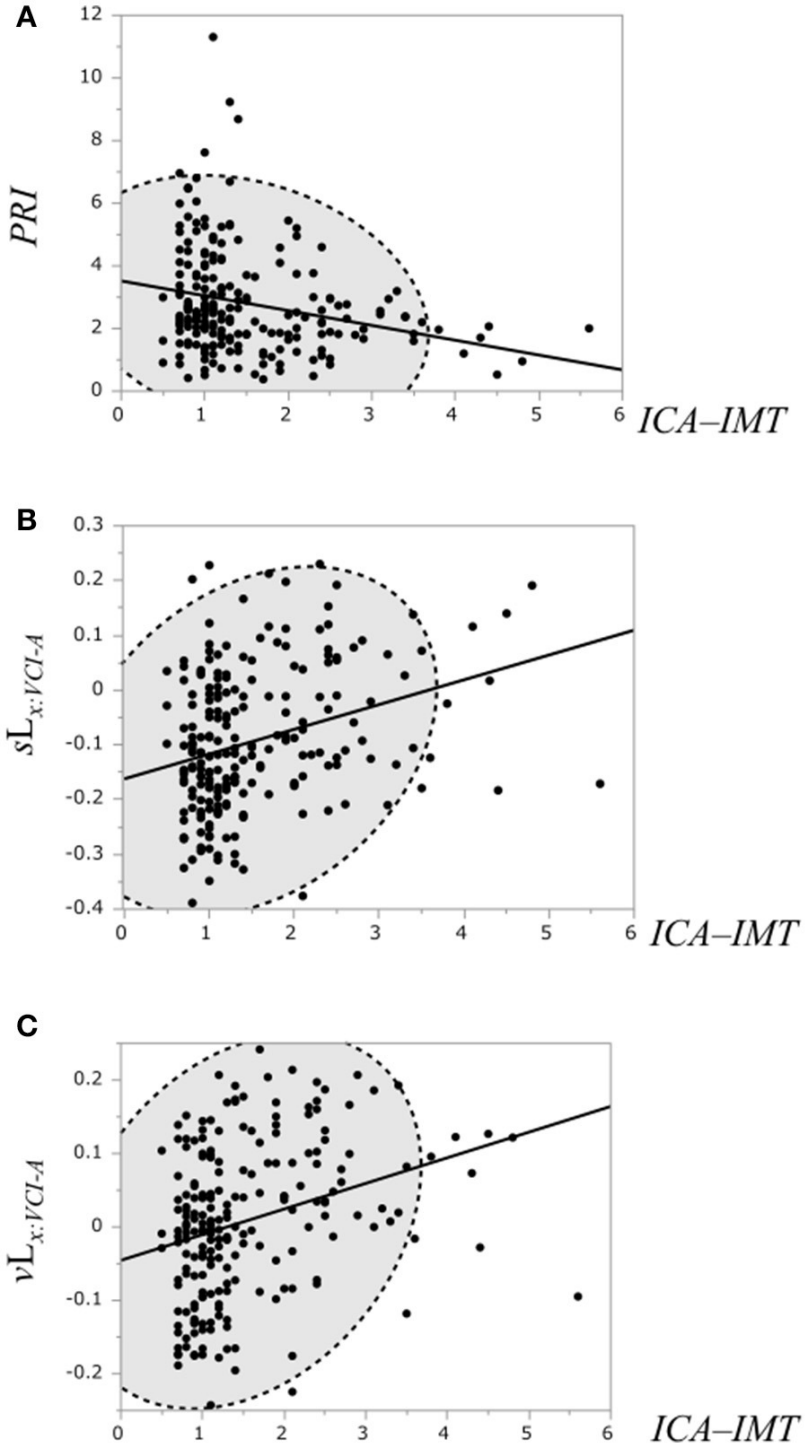
**(A)** Scatterplot of the PRI and the ICA–IMT. **(B)** Scatterplot of the binary likelihood *s*L_*x*:*VCI*−*A*_ and the ICA–IMT. **(C)** Scatterplot of the binary likelihood *v*L_*x*:*VCI*−*A*_ and the ICA–IMT. Broken elliptical shape indicates a 95% confidence ellipse for normally distributed data.

The correlation of the ICA–IMT and the PRI, the binary likelihood *s*L_*x*:*VCI*−*A*_ and the binary likelihood *v*L_*x*:*VCI*−*A*_ was examined, respectively (Figure 7). Linear regression analysis showed a weak negative correlation between the ICA–IMT and the PRI (*r* = −0.25, *P* = 0.0002), and a weak positive correlation between ICA–IMT and the binary likelihoods *s*L_*x*:*VCI*−*NLc*_ (*r* = 0.31, *P* < 0.0001) and *v*L_*x*:*VCI*−*NLc*_ (*r* = 0.3, *P* < 0.0001).

## Discussion

In the present study, a relationship between subclinical atherosclerosis and EEG markers of PRI, *s*NAT, *v*NAT in an elderly population was investigated, respectively. In results, we identified 2 important findings. First, it was confirmed that EEG spectrum intensity (sNAT) and spectrum steepness (vNAT) are related to pathological changes indicative of subclinical carotid atherosclerosis. In particular, the binary likelihoods *s*L_*x*:*VCI*−*A*_ and *v*L_*x*:*VCI*−*A*_ in group D (ICA–IMT, ≥1.9 mm) suggests a risk of an EEG pathological change. Second, NAT might visually show topographic information about EEG alterations in the elderly with increased ICA–IMT. Although there was difficult to interpret an association between all the 210-dimensional NAT spaces and EEG alterations regarding subclinical atherosclerosis, the characteristics distributions of sNAT and vNAT in the elderly with increased ICA–IMT might have appeared at a specific frequency range (e.g., 7.8 Hz). As far as EEG alterations of a dominant parieto-occipital alpha rhythm goes, in sNAT, there were decreased activities over occipital areas at alpha frequency range of 10.9 Hz, and increased activities over occipital areas at theta frequency range of 7.8 Hz, suggesting the alterations of EEG spectrum intensity from alpha to upper theta ranges. Similarly, in vNAT, there were decreased activities over occipital areas at alpha frequency range of 9.4 Hz and increased activities over occipital areas at theta frequency range of 7.8 Hz, suggesting the alterations of EEG spectrum steepness, that is, more spectrum blur (i.e., undersynchrony) at upper theta frequency range and more spectrum sharpness (i.e., oversynchrony) at alpha frequency range.

There were many quantitative EEG markers of PRI, EEG global power, an asymmetry-based EEG marker, and NAT for detecting mild cognitive impairment (MCI). Previous findings in patients with MCI secondary to ischemic vascular damage, have demonstrated an increase in low frequency power and a decrease in high frequency power (Nagata, 1988; Nagata et al., 1989). Other studies of subjects with cognitive decline have identified an increase in theta relative power and a decrease in gamma relative power (Moretti et al., 2007a,b, 2009). Recently, Sheorajpanday et al reported about EEG global power in subcortical VCI, no dementia independently predicts vascular impairment, and brain symmetry index reflects severity of cognitive decline (Sheorajpanday et al., 2014). Although in this study, NAT was not compared with other markers of EEG global power or an asymmetry-based EEG marker, we have previously demonstrated a decrease of the PRI in VCI patients compared with normal controls, namely an increase in low frequency power and a decrease in high frequency power (Shibata et al., 2014). In the present study, we confirmed a decrease of the PRI in severe atherosclerosis group D compared with normal control group A. Further investigations are needed in order to widen the clinical applicability of EEG markers, that is, what is a best practical and cost-effective analysis for detecting MCI among many EEG markers of PRI, EEG global power, asymmetry-based EEG marker, NAT (sNAT, vNAT), and so on.

Carotid atherosclerosis might act as a marker of intracerebral and generalized atherosclerosis and small vessel disease, and has been associated with increased cognitive decline. Some studies have suggested that stenosis of the ICA may be an independent risk factor for cognitive impairment (Rao, 2001; Johnston et al., 2004; Mathiesen et al., 2004). High-grade stenosis of the ICA may be associated with MCI, even without evidence of infarction on MRI (Sztriha et al., 2009). In a large cohort study, high-grade stenosis was seen as an important predictor of cognitive decline (Johnston et al., 2004). Several population-based studies of elderly subjects (aged > 65 years) have found associations between carotid IMT and subsequent cognitive decline (Haan et al., 1999; Sander et al., 2010). However, the pathophysiology of VCI in carotid atherosclerosis without evidence of infarction on MRI is unclear (Mathiesen et al., 2004). In a previous study using magnetoencephalography (MEG), a theta rhythm (6–8 Hz) over parieto-temporal areas, which was separated from a occipital alpha rhythm, appeared in patients with internal carotid artery occlusive disease (Seki et al., 2005). Although conventional EEG in general may not be suitable to separate the upper theta rhythm of 6–8 Hz from the occipital alpha rhythm of 8–12 Hz, NAT might detect the increase of the upper theta frequency bands (more precisely, from 6.3 to 7.8 Hz) and the decrease of the alpha frequency bands over temporo-occipital areas in the elderly with increased atherosclerosis, which of regions might be in part corresponding to the characteristic findings of parieto-temporal upper theta activity (6–8 Hz) measured by MEG (Seki et al., 2005). Although another EEG study (Hsiao et al., 2016) pointed out that a carotid stenosis <50% did not alter theta (4–8 Hz) oscillations, the present study suggests that, at a specific frequency band from alpha to upper theta range, a subtle EEG alternation might appear in the elderly at an early stage of atherosclerosis (ICA–IMT ≥ 1.9 mm) before misery perfusion. Therefore, EEG markers included MEG might be more useful for detecting subtle cognitive decline, rather than MRI and conventional visual EEG analysis. To detect subtle cognitive decline with alternation of EEG, prospective studies are needed to investigate the precise association between EEG markers included MEG and several neuropsychological tests (e.g., Wechsler Adult Intelligence Scale -III, Raven's progressive matrices, Rey–Osterrieth complex figure test, and Montreal Cognitive Assessment) (Larner, 2012; Kirkpatrick et al., 2014; Sheorajpanday et al., 2014).

In general, EEG oscillations in the alpha and theta band reflect cognitive and memory performance (Klimesch, 1999). A recent study confirms the major role of the interplay of theta (5–7 Hz) and alpha (8–12 Hz) frequency in the cognitive impairment, that is, the local compensation in the baseline activity at a theta and alpha frequency range (Abuhassan et al., 2014). The structure of the model suggests that cortical oscillations respond differently to compensation mechanisms in the cognitive impairment. In the present study, changes of characteristics distributions regarding sNAT in the elderly with increased IMT have appeared at specific frequency ranges (anchor frequencies) of theta (7.8 Hz) and alpha (10.9 Hz), suggesting an insufficient interplay between a theta and alpha frequency band in the Default Mode Network (DMN). Similarly, changes of characteristics distributions regarding vNAT in the elderly with increased IMT have appeared at specific frequency ranges of theta (7.8 Hz) and alpha (9.3 Hz) (anchor frequencies), suggesting that disruptions of a balance in the DMN might be projected on the mapping of vNAT through a spectrum steepness on EEG.

Although NAT might detect abnormal cortical neuronal activity in VCI patients, our results should be interpreted with caution based on the following limitations. One limitation is that it seems to be very far away from understanding a concept and getting a clinical merit of sNAT and vNAT markers. It is easier to understand a concept of sNAT than that of vNAT, because sNAT abnormality in the power partition over the spectrum of EEG signals (i.e., hyperspectrum intensity or hypospectrum intensity) is partially similar to PRI (see Supplementary Material). On the other hand, it is likely to confuse a concept of vNAT characterized by a ratio of power spectrum between the adjacent power component, with a well-known EEG coherence which indicating the spectral correlation between electrodes. Although, at this time, we have no obvious findings about the relation between vNAT and the well-known EEG coherence, Musha thought that the coherence causes spiky variations of the PS, suggesting a relationship between EEG coherence and spectrum steepness in vNAT. When vNAT is larger or smaller than that of the NLc, the collective neuronal activities are in the undersynchrony or oversynchrony, in other words, power spectrum blur or power spectrum sharpness, respectively. In case of flat variations or gentle gradient of PS (i.e., power spectrum blur), the collective randomly activated neurons have no modulation and no meaningful biological signals are transmitted, because the neuronal activities are generated at random. In case of spiky variations or steep gradient of PS at a specific frequency band (i.e., power spectrum sharpness), the collective neuronal activities are partly coherent and partly random, and some signal contents are transmitted through the collective neuronal activities. However, not to confuse vNAT with the well-known EEG coherence, we would like to change a mode of expression from the EEG coherence to EEG spectrum steepness regarding vNAT in this paper. Therefore, a precise relationship between vNAT marker and EEG coherence needs to be investigated. Second, EEG analysis is characterized by low spatial resolution (several centimeters) when compared to structural MRI. However, NAT includes 10 frequency bands ranging from 4.7 to 18.8 Hz, which might convey peculiar physiological information on cortical activity beyond MRI. Therefore, we would like to investigate the integration of brain structure based on MRI with brain function based on EEG at a specific frequency range (e.g., 7.8 Hz). Anatomical MRI and functional EEG might provide complementary information into the process of MCI in future.

The prevention of carotid atherosclerosis could protect against vascular cognitive decline, but properly designed intervention studies are needed to demonstrate whether treatment of carotid atherosclerosis could lower the risk of cognitive decline in people without prior cerebrovascular disease. In previous studies, the highest quintile of carotid IMT was associated with dementia risk (Van et al., 2007; Wendell et al., 2012). Although ICA–IMT must reach a criterion thickness to become predictive of dementia from large-scale epidemiologic investigations, the present study did not prove that the abnormality of NAT is an independent risk factor for cognitive decline in future. Therefore, longitudinal studies are needed to examine these associations between EEG markers and ICA–IMT, and neuropsychological tests in the elderly with subclinical carotid atherosclerosis. In a first checkup for cognitive decline in community-dwelling elderly people, a combination with neuropsychological test, ultrasonography, and EEG might be useful for screening subclinical cognitive impairment.

Electroencephalographic (EEG) analysis has practical advantages for neuronal activity assessment because it is inexpensive, portable, noninvasive, sensitive to MCI, and it provides direct, real-time physiological information regarding 4–8 Hz (theta), 8–13 Hz (alpha), 13–30 Hz (beta) frequency range. To widen the applicability of EEG for detecting MCI, reliable, standardized, and user-friendly methods should be needed and developed. NAT could provide information on pathophysiology at a specific frequency band, and asymmetrical visual information of cortical neuronal activity to assess cognitive decline. The present study suggests that physiological aging accompanied by EEG alpha (8–13 Hz) decreasing (Rossini et al., 2007) and MEG theta (6–8 Hz) increasing over the posterior parietal and occipital areas (Puligheddu et al., 2005) might be affected by a increased atherosclerosis to some extent. Bamidis have proposed the neuroscience of physical and cognitive interventions in aging (Bamidis et al., 2014). Further improvements in the NAT system are needed to detect prior cognitive decline in aging and support a monitor of physical and cognitive interventions to maintain a healthy brain.

## Conclusion

Community-dwelling elderly subjects in the upper quintile for ICA–IMT (≥1.9 mm) were at greater risk of an EEG change reflecting VCI as assessed by NAT. Our study highlights the importance of early intervention for carotid atherosclerosis to minimize the risk of an EEG change that might be related to subsequent VCI.

## Ethics statement

The ethical committees in the University of Tsukuba. All participants signed a consent form approved by the ethical committees in the University of Tsukuba.

## Author contributions

The authors contributed to this manuscript in the following manner: study design (TS, TM, YK, HM, and TS), data acquisition and analysis (YK, MT, HM, and KN), interpretation of results (MK, YH, NK, and SK). All authors contributed to revise and approve the final version of the manuscript and agree to be accountable for this work.

### Conflict of interest statement

The authors declare that the research was conducted in the absence of any commercial or financial relationships that could be construed as a potential conflict of interest.

## Supplementary material

Supplementary Figure1 Two normalizations for understanding sNAT figuratively. Normalization of sNAT is often likened to a photograph processing. **(a)** PS, **(b)** NPS, **(c)** sNAT corresponds to **(d–f)**, respectively. **(d)** means a original subject (e.g., ship) in a frame, **(e)** means the adjustment of the size to frame the subject, and **(f)** means that the centroid of the subject is moved to the center of the frame.
