# Supplementary figures and images for: Altered Neuronal Activity Topography Markers in the Elderly with Increased Atherosclerosis
Source: Front Aging Neurosci. 2017 Jul 6;9:216. doi: 10.3389/fnagi.2017.00216 (PMC5498522; doi:10.3389/fnagi.2017.00216)

## Slide 1
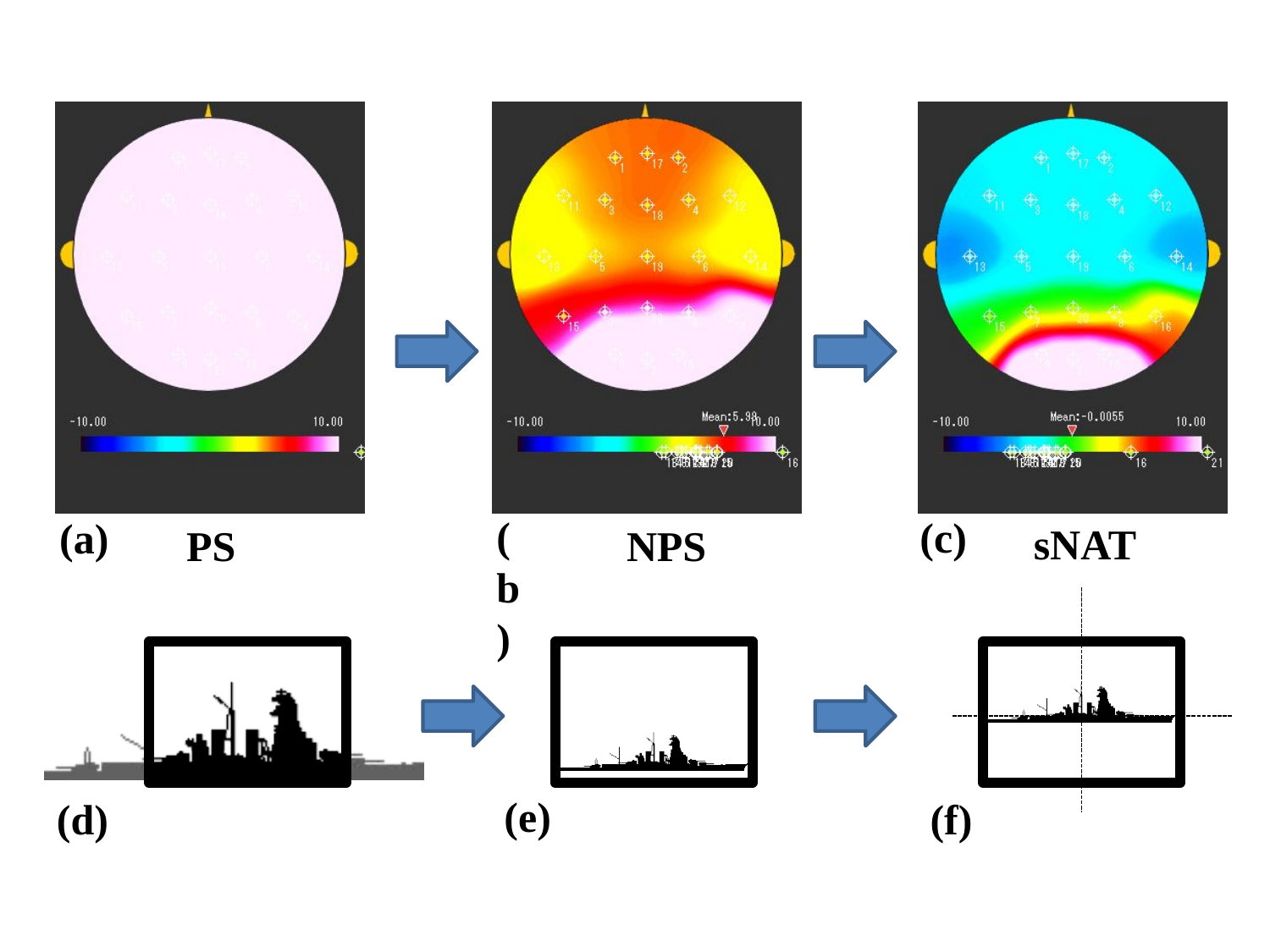

(b)
(c)
(a)
sNAT
PS
NPS
(e)
(d)
(f)

Supplement: Supplementary Figure1 — Two normalizations for understanding sNAT figuratively. Normalization of sNAT is often likened to a photograph processing. (a) PS, (b) NPS, (c) sNAT corresponds to (d–f), respectively. (d) means a original subject (e.g., ship) in a frame, (e) means the adjustment of the size to frame the subject, and (f) means that the centroid of the subject is moved to the center of the frame. [file Presentation1.PPTX]
